# Role of Sirtuins in the Hallmarks of Skin Aging

**DOI:** 10.1111/acel.70789

**Published:** 2026-11-05

**Authors:** Rishabh Kala, Antonella Rella, Whitby Eagle, Earl Goyarts, Jian Cao, Binwei Deng, Kelly Dong, Chloe Schmit, Matthew Cappock, Krystle Corallo, Jacqueline Trivero

**Affiliations:** ^1^ The Estée Lauder Companies Melville New York USA; ^2^ China Innovation Labs The Estée Lauder Companies Shanghai China

**Keywords:** dermal fibroblasts, extracellular matrix, genomic instability, hallmarks of aging, keratinocytes, senescence, sirtuin, skin aging

## Abstract

Sirtuins are a family of enzymes that regulate cell homeostasis through pathways impacting genomic stability, mitochondrial function, cellular senescence, inflammation, and autophagy. In the skin, these pathways protect against extrinsic insults, maintain keratinocyte stem cells, and contribute to the overall structure and integrity of the skin barrier. Sirtuins decline with chronologic age, with UV exposure also impacting their activity, resulting in a loss of this homeostasis. There are 13 established biological hallmarks of aging. This review presents the current evidence of the mechanistic roles of sirtuins for each of these hallmarks, particularly in relation to skin.

## Graphical abstract

In the skin, sirtuins support the integrity of the epidermis and dermis and protect the skin from environmental insults by maintaining cellular homeostasis. Sirtuins decline with chronological age, contributing to the biological hallmarks of aging, with increasing evidence emerging of their specific roles in skin aging.

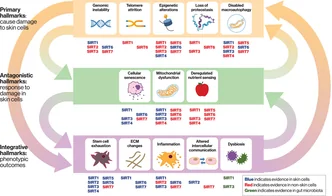

## 1 Introduction

Healthy skin is comprised of a self‐renewing epidermal layer and an inner dermal layer of fibroblasts embedded in a complex extracellular matrix (ECM). These layers provide a protective barrier with elasticity, smooth texture, and plumpness (Furman et al. 2025). Skin aging is attributed to intrinsic factors of chronological age and genetics, and extrinsic factors including air pollution, smoking, and ultraviolet radiation (UVR) (Fan et al. 2024).

Thirteen molecular mechanisms have canonically been ascribed as “Hallmarks of Aging” (Kroemer et al. 2025). The complex interplay of these hallmarks in the skin contributes to a progressive breakdown in cellular homeostasis and dysregulation of repair mechanisms, which manifest visibly as wrinkles, sagging skin, and aberrant pigmentation (Furman et al. 2025). Sirtuins are a family of enzymes integral to maintaining cellular homeostasis, particularly in response to UVR. Here, we review published evidence linking the cellular activities of sirtuins in the skin to their impact on these hallmarks of aging.

## 2 SIRT1‐7: Location and Primary Function

First discovered in *Saccharomyces cerevisiae* , sirtuins are evolutionarily conserved, with seven enzymes identified in mammals (Michan and Sinclair 2007). Their primary enzymatic activity is nicotinamide adenine dinucleotide (NAD+)‐dependent deacetylation; however, sirtuins demonstrate multiple functions, acting on multiple substrates (Table 1) (Grabowska et al. 2017; Vassilopoulos et al. 2011). Several sirtuins move between cellular locations in response to certain conditions (e.g., mitosis, DNA damage) and carry out disparate functions in these locations (Grabowska et al. 2017; Vassilopoulos et al. 2011).

**TABLE 1:** Sirtuins: Location and functions.

Sirtuin	Location in cell	Enzymatic function	Cellular functions	References
SIRT1	Nucleus Translocates to cytoplasm in response to growth factor decline or inhibition of PI3K/Akt pathway	Deacetylase Deacylase	DNA repair: SIRT1 interacts with sites of DNA damage and recruits DNA repair machinery in MEF, including the repair protein NBS‐1, a component of the DNA damage sensor complex MRN, and Rad51, part of the DSB HR process. SIRT1 deacetylates XPG A, an essential factor in the NER pathway. Telomere stabilization: SIRT1 binds to telomeric repeats, deacetylating the RecQ helicase WRN to protect from digestion and facilitate telomere replication; SIRT1 deacetylates c‐Myc enhancing telomerase transcription. Transcription regulation: SIRT1 deacetylates multiple histones to stabilize the heterochromatin structure and regulate expression of transcription factors. Mitochondrial biogenesis: SIRT1 regulates PGC‐1α pathway and synthesis of ETC components and their transport from the nucleus to the mitochondria. Mitochondrial metabolism: Regulates glucose metabolism through AMPK, activates PGC‐1α and inhibits FOXO1 to regulate transcription of target genes for gluconeogenesis and FAO. Oxidative stress response: SIRT1 regulates expression of antioxidant defense mechanisms. Mitochondrial dynamics: SIRT1 promotes fission through activation of the SIRT1/AMPK/protein kinase A (PKA)/DRP1 pathway. Autophagy: Promotes mitophagy and autophagy through activation of FOXO1 and FOXO3 and AMPK/mTOR pathways. Keratinocyte differentiation: SIRT1 regulates proliferation and differentiation, through calcium‐dependent signaling. Cell proliferation: Deacetylates LC3 and retinoblastoma, regulates expression of transcription factors to promote proliferation; regulates AMPK/mTOR and STAT3 for antiproliferative effects. Inflammation: Regulation of innate and adaptive immune response.	Vassilopoulos et al. (2011), Wu et al. (2022), Grabowska et al. (2017), Fan and Luo (2010), Kim et al. (2022), Oberdoerffer et al. (2008), Yuan et al. (2007), Palacios et al. (2010), Wątroba et al. (2017), Xu et al. (2023), Lagunas‐Rangel (2025), Blander et al. (2009), Gámez‐García and Vazquez (2021), and Michan and Sinclair (2007)
SIRT2	Cytoplasm Translocates to nucleus to regulate cell cycle	Deacetylase Deacylase Demyristoylase	Maintains cellular structure: SIRT2 deacetylates α‐tubulin and HDAC6 to modulate the cytoskeleton. Cell‐cycle control: Regulates chromatin condensation through deacetylation of H4K16 during G2/M transition. Cell proliferation: Regulates Myc to promote proliferation. Barrier function: SIRT2 regulates Claudin‐4, which impacts tight junction between cells. Oxidative stress response: SIRT2 activates Nrf2, and SOD expression through FOXO3. Inflammation: Anti‐inflammatory through deacetylating p63 subunit of NF‐κB; may have pro‐inflammatory effects. Cell differentiation: SIRT2 can impact adipocyte differentiation through modification of FOXO1. Mitochondrial metabolism: SIRT2 deacetylates PEPCK to activate gluconeogenesis in low nutrient conditions. Mitochondrial dynamics: SIRT2 regulates fission through the MEK1‐ERK‐DRP1 and AKT1‐DRP1 axes. Mitophagy: Negative regulator that impairs mitophagy through deacetylation of autophagy protein 5 (ATG5), interfering with the formation and elongation of autophagosomes that engulf and degrade damaged mitochondria. Autophagy: SIRT2 binds and regulates TFEB expression, regulating lysosomal biogenesis and autophagosome formation.	Vassilopoulos et al. (2011), Grabowska et al. (2017), Wu et al. (2022), Wang, Meyer, et al. (2019), Miwa et al. (2025), North et al. (2003), Serrano et al. (2013), Gámez‐García and Vazquez (2021), Silva et al. (2017), and Liu et al. (2017)
SIRT3	Nucleus Mitochondria	Deacetylase Lysine decrotonylase	Genomic stability: Nuclear, full‐length SIRT3 keeps chromatin compressed and represses transcription. DNA repair: SIRT3 translocates from the mitochondria to the nucleus in response to DNA damage, where it colocalizes with p53‐binding protein‐1, facilitating NHEJ repair. Mitochondrial biogenesis: SIRT3 deacetylates FOXO3, upregulating PGC‐1α and TFAM to activate synthesis of ETC components and their transport from the nucleus to the mitochondria. Mitochondrial metabolism: SIRT3 regulates FAO, ETC activity, ATP production, glucose oxidation, ketone body production, and the urea cycle. Mitochondrial dynamics: SIRT3 deacetylates OPA1 to induce fusion or FOXO3 to induce fission. Oxidative stress response: SIRT3 increases expression and activation of SOD2. Mitophagy: SIRT3 promotes mitophagy through activation of FOXO3, AMPK/mTOR pathways and PINK1/Parkin deacetylation. Proteostasis: SIRT3 regulates the mitochondrial unfolded protein response. Keratinocyte stem cell maintenance: SIRT3 modulates mtSOX and prevents expression of differentiation markers. Cell proliferation: Deacetylates SHMT2 to support proliferation.	Trinh et al. (2024), Bao et al. (2014), Zhou et al. (2022), Grabowska et al. (2017), Tao et al. (2010), Scher et al. (2007), Sengupta and Haldar (2018), Kumar and Lombard (2015), Wu et al. (2022), Hirschey et al. (2010), Xu et al. (2023), Lagunas‐Rangel (2025), Papa and Germain (2014), and Bause et al. (2013)
SIRT4	Mitochondria	Deacetylase ADP‐ribosyl transferase Lipoamidase Deacylase Weak deacetylase	Mitochondrial metabolism: SIRT4 suppresses GDH activity, inhibits FAO, enhances ATP production. Oxidative stress response: Reduces ROS by inhibiting HIF‐1α and activating Akt/mTOR pathway. Mitochondrial dynamics: SIRT4 deacetylates OPA1 to promote fusion, thereby reducing fission and mitophagy. Mitophagy: Negative regulator of autophagy via activation of the Akt/mTOR pathway, and disrupts tagging of dysfunctional mitochondria. Cell proliferation: Suppresses proliferation by inhibiting glutamine metabolism.	Wu et al. (2022), Kumar and Lombard (2015), Huang et al. (2023), Mathias et al. (2014), Pannek et al. (2017), Xu et al. (2023), He et al. (2022), and Lagunas‐Rangel (2025)
SIRT5	Mitochondria	Weak deacetylase Deacylase Desuccinylase Demalonylase Deglutarylase	Mitochondrial metabolism: Regulates glycolysis, TCA cycle, ETC, FAO, ketone body production, and the urea cycle. Oxidative stress response: SIRT5 activates SOD1. Mitochondrial dynamics: SIRT5 decreases fission. Mitophagy: SIRT5 can increase or decrease mitophagy depending on the pathway and activator, notably through its interaction with PINK and Parkin. Cell proliferation: Supported through increasing MAPK activity and regulating expression of transcription factors; suppresses cell proliferation via ERK/STAT pathway. Inflammation: Suppressed via ERK/STAT pathway.	Park et al. (2013), Kumar and Lombard (2018), Grabowska et al. (2017), Wang, He, and Shi (2023), Du et al. (2011), Tan et al. (2014), Nakagawa et al. (2009), Xu et al. (2023), Nishida et al. (2015), and Wu et al. (2022)
SIRT6	Nucleus	Deacetylase ADP‐ribosyl transferase Deacylase	Transcription regulation: SIRT6 deacetylates multiple histones to stabilize the heterochromatin structure and regulate expression of transcription factors. Genome stability/DNA repair: SIRT6 is integral in multiple DNA repair processes, including stabilizing the DSB protein‐kinase repair factor, HR, NHEJ, and BER. Telomere integrity: SIRT6 deacetylates telomeric histones and activates DNA repair mechanisms, enabling recruitment of telomere processing factors, including WRN helicase, to prevent the loss of telomeres. SIRT6 recruits the chromatin remodeling protein SNF2h, to facilitate the decondensation of chromatin and allow access for DNA repair machinery. SIRT6 enhances expression of the telomerase component (TERT) in human cells. Cell‐cycle regulation: SIRT6 silences heterochromatin to facilitate correct mitosis; stabilizes spindle formation and chromosome alignment. Metabolism regulation: SIRT6 controls the expression of numerous glycolytic, gluconeogenesis, lipid metabolism, and insulin‐signaling genes. Mitochondrial biogenesis: SIRT6 increases AMPK and PGC‐1α activity and/or reduces the activity of Sox6. Oxidative stress response: SIRT6 interacts with Nrf2, which regulates the expression of antioxidant genes. Inflammatory response: Regulates TNF‐α secretion and is a negative regulator of NF‐κB. Mitophagy: Indirectly regulates mitophagy through stimulation of AMPK, which then increases FOXO3 activity. Cell proliferation: Promoted by repressing AMPK signaling, activating ERK.	Chang et al. (2020), Toiber et al. (2013), Wei et al. (2024), Wu et al. (2022), Grabowska et al. (2017), Garcia‐Peterson, Wilking‐Busch, et al. (2017), Li et al. (2021), Wątroba et al. (2017), McCord et al. (2009), Toiber et al. (2013), Chen et al. (2017), Chen et al. (2020), Michishita et al. (2008), Gao et al. (2018), Chen et al. (2021), Zhong et al. (2010), Xu et al. (2023), Gámez‐García and Vazquez (2021), and Lagunas‐Rangel (2025)
SIRT7	Nucleus and nucleolus	Deacetylase Desuccinylase ADP‐ribosyltransferase Debutyrylase Deglutarylase Decrotonylase	Genomic integrity: SIRT7 deacetylates H3K18, H2A and H2B to transiently compact chromatin at the site of DNA damage to prevent DNA digestion, and then permits access when DNA machinery arrives. Transcription regulation: Regulation of rRNA transcription and ribosome biogenesis. DNA repair: SIRT7 translocates from the nucleosome to sites of DNA damage in the nucleus in a PARP‐dependent manner, concomitantly reducing its chromatin association and increasing access to repair machinery for HR and NHEJ. Nucleolar stress response: Under steady‐state, SIRT5 keeps chromatin condensed and maintains rDNA integrity. Binds to and stabilizes p53. Metabolic stress response: During caloric restriction, SIRT7 promotes gluconeogenesis. Inflammatory cell regulation. Mitophagy: Negative regulator that deacetylates Parkin, and inhibits mTOR.	Kumari et al. (2021), Ianni et al. (2024), Grabowska et al. (2017), Wu et al. (2022), Raza et al. (2024), Ford et al. (2006), Zhang et al. (2016), Vazquez et al. (2016), Gámez‐García and Vazquez (2021), and Lagunas‐Rangel (2025)

Abbreviations: BER, base excision repair; ERK, extracellular signal‐regulated kinase; ESC, embryonic stem cells; ETC, electron transport chain; FAO, fatty acid oxidation; FOXO, Forkhead box protein of class O; GDH, glutamate dehydrogenase; HDAC6, Histone deacetylase 6; HIF‐1, hypoxia‐inducible factor 1; HR, homologous repair; MEF, mouse embryonic fibroblasts; MEK1, mitogen‐activated protein kinase kinase‐1; MRN, Mre11‐Rad50‐Nbs1; NBS, Nijmegen Breakage Syndrome; NER, nucleotide excision repair; NHEJ, non‐homologous end joining; NLRP3, nucleotide‐binding oligomerization domain‐like receptor (NLR) family pyrin domain containing 3; NRF2, nuclear factor erythroid 2‐related factor 2; PEPCK1, phosphoenolpyruvate carboxykinase; SHMT2, serine hydroxymethyltransferase 2; SOD, superoxide dismutase; TERT, telomerase reverse transcriptase; TET2, ten‐eleven translocation 2; TFEB, transcription factor EB; UVR, ultraviolet radiation; XPG, Xeroderma pigmentosum group.

Sirtuins maintain cellular homeostasis through the regulation of transcription via histone deacetylation, DNA repair, metabolism, oxidative stress response, inflammation, and autophagy (Wu et al. 2022; Zhou et al. 2022; Rubinsztein et al. 2011). Located in the nucleus, SIRT1 and SIRT6 maintain genomic stability through DNA repair and telomere protection (Oberdoerffer et al. 2008; Michishita et al. 2008). SIRT1 is also involved in the regulation of multiple histone and nonhistone substrates, affecting glucose homeostasis, mitochondrial biogenesis, inflammation, apoptosis, and stress resistance (Poulose and Raju 2015). Its nuclear location appears to be maintained by the PI3K/Akt pathway, as studies in nonhuman/non‐skin models suggest it is shuttled to the cytosol in aging and senescent cells (Meng et al. 2011; Tanno et al. 2007). SIRT2 resides in the cytoplasm under basal conditions, translocating to the nucleus to participate in cell cycle regulation (Grabowska et al. 2017). Inactive SIRT3 resides in the nucleus and is later translocated to the mitochondria, where it is activated; however, it is also recruited to sites of DNA damage, suggesting its translocation back to the nucleus for this function (Sengupta and Haldar 2018).

The mitochondrial sirtuins (SIRT3, SIRT4, SIRT5) regulate metabolism, ATP production, and energy stores in response to nutrient availability (Wu et al. 2022). SIRT7 is the only sirtuin located in the nucleolus, where it regulates rDNA transcription, ribosome biogenesis, and the nucleolar stress response (NSR) (Kumari et al. 2021). These roles have been elucidated across species, cell and organ types, and in disease.

## 3 Role of Sirtuins in Skin

Sirtuin functions specific to skin include maintaining the barrier and ECM integrity, regulating keratinocyte differentiation, protection from environmental insults, and facilitating repair. Undifferentiated keratinocyte stem cells reside in the basal layer of the epidermis where they proliferate and differentiate to regenerate the epidermis, as needed. Several sirtuins participate in the regulation of keratinocyte differentiation. Overexpression of SIRT1 or SIRT2 increases differentiation of keratinocytes while reducing proliferation; in contrast, SIRT3 activity decreases mitochondrial ROS (mtROS) and thus suppresses keratinocyte differentiation (Blander et al. 2009; Ming et al. 2014; Bause et al. 2013). SIRT2 also directly contributes to keratinocytes' paracellular barrier function by deacetylating the tight junction protein claudin‐4 (Miwa et al. 2025).

Within the dermal layer, SIRT1 and SIRT6 prevent the breakdown of collagen (see section 6.10 Extracellular Matrix Changes), while SIRT2 deacetylates α‐tubulin to maintain cytoskeletal organization and cell shape of fibroblasts (Orioli and Dellambra 2018; North et al. 2003).

Cutaneous wound repair involves several stages: inflammation, proliferation, migration, maturation, and remodeling. Dermal fibroblasts migrate to the site to deposit collagen into the ECM. The effects of sirtuins on dermal repair processes have been partially elucidated. SIRT1 downregulates inflammatory mediators, including NF‐κB, IL‐6, and TNF‐α, while activating fibroblast migration to the site of insult and impacting collagen density (Gilbert et al. 2023). Decreased SIRT3 expression, seen in the skin of diabetic patients, appears to result in a decline in skin fibroblast migration (Gilbert et al. 2023). SIRT6 inhibition in the dermis increases NF‐κB mediators and oxidative stress (Wu et al. 2022). In other wound‐healing models, miR‐125b appears to inhibit SIRT7, increasing fibroblast migration (Gilbert et al. 2023; Xia et al. 2022).

Sirtuins have a key role in protecting and repairing the skin in response to UV radiation (Figure 1). Both UVA and UVB increase ROS and induce DNA damage, activating stress‐response pathways including JNK and p53. The majority of sirtuins are initially downregulated upon UV exposure, followed by more complex, sirtuin‐specific adaptive responses. When first exposed, SIRT1 mRNA and protein levels decreased dose‐dependently in human skin biopsies of middle‐aged female subjects compared with matched non‐exposed subjects (Ding et al. 2018), an effect driven by ROS‐mediated JNK signaling in keratinocytes (Cao et al. 2009). However, SIRT1 acts as a negative regulator of JNK signaling, possibly via deacetylation of upstream substrates. Additionally, SIRT1 negatively regulates UV‐induced p53 acetylation, thereby reducing apoptosis and acting as a positive activator of AMP‐activated protein kinase (AMPK) (Cao et al. 2009). SIRT3 and SIRT4 have a temporal, inverse pattern of expression and activity in human keratinocytes (Dong et al. 2012). Noncytotoxic doses of UVB initially suppress SIRT3 and SIRT4; however, SIRT4 rebounds within hours to approximately 20% above non‐irradiated skin due to UV‐induced decreases in microRNA (miRNA)‐15b, while SIRT3 levels remain low, resulting in a net decrease in ATP production, and disruption of their inverse temporal expression pattern (Lang et al. 2016; Dong et al. 2012). The exact mechanism by which SIRT3 declines and SIRT4 rebounds remains undetermined; however, in response to DNA damage, decreased NAD+ would have a greater impact on SIRT3, as it has the strongest NAD+‐dependent deacetylase activity (Wei et al. 2024), while DNA damage increases the protein kinases ATM (ataxia telangiectasia mutated) and ATR (ataxia telangiectasia and Rad3‐related), which in turn upregulate SIRT4 (Wątroba et al. 2017). ROS accumulates due to SIRT4 inhibiting SIRT3‐mediated MnSOD (superoxide dismutase) activation (Betsinger and Cristea 2019). In addition, UVB decreases SIRT3 and SOD2 in human epithelial cells (skin keratinocytes or oral epithelial cells) (Gan et al. 2023). In vivo, UVB‐exposed rat skin showed reduced SIRT3, SOD, and collagen‐I, while ROS, p38/JNK signaling, and matrix metalloproteinases (MMPs) were elevated (Mu et al. 2021). SIRT5 expression is dysregulated after UV exposure, as biopsies of UV‐exposed neck skin showed SIRT5 expression was lower than non‐exposed skin in young subjects, but was profoundly decreased in midlife and elderly subjects (Zhang et al. 2026). It should be noted that one study found no change in SIRT5 expression in human dermal fibroblasts (HDFs) after UVR; however, the donors' age was not reported (Lee, Shin, Kim, Kim, et al. 2021). SIRT6 can be upregulated by UVB 24 h after exposure via PI3K/Akt in normal human epidermal keratinocytes (NHEK) (Ming et al. 2014). A study of mice exposed to increasing UVB doses over 10 weeks showed significant increases in SIRT6 in the upper layers of the epidermis, with SIRT6 located in the cytoplasm rather than its typical location in the nucleus (Oh et al. 2022). In HDFs, SIRT6 was suppressed via upregulation of miR‐663a and miR‐4706, which target its 3′UTR. Overexpression of these miRNAs results in SIRT6 silencing, thereby promoting HDF senescence, DNA damage, and ROS accumulation (Li et al. 2025). In contrast, SIRT7 activity increases post‐UVR, stabilizing p53 in human keratinocytes and in vivo mice, and causing cell cycle arrest (Ianni et al. 2021; Lagunas‐Rangel 2022; Kumari et al. 2021). UVR downregulates SIRT2 and SIRT3 expression in HDFs, though the decline in SIRT2 was only 10%–20% (Lee, Shin, Kim, Kim, et al. 2021; Lee, Shin, Kim, Park, et al. 2021). Through epigenetic regulation of DNA repair and telomere protection, SIRTs 1, 6, and 7 help maintain genomic integrity under UV stress (Orioli and Dellambra 2018; Gilbert et al. 2023). With prolonged or repeated exposure, these mechanisms decline, causing senescence, collagen and elastin breakdown, and dermal structure loss, with visible signs of photoaging including wrinkles and texture changes (Flament et al. 2013).

**FIGURE 1:**
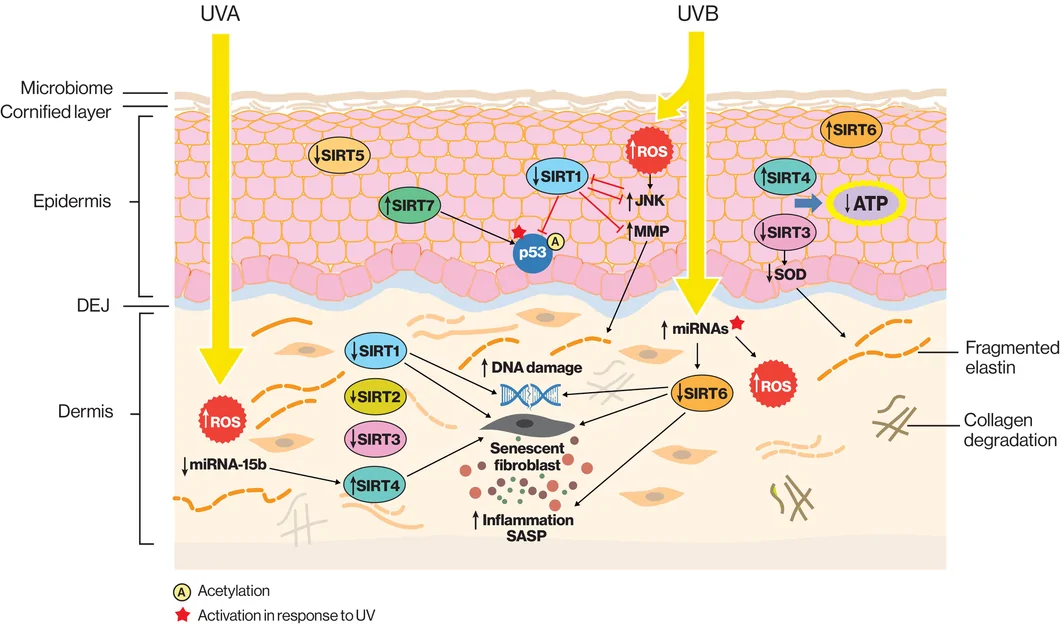
Effects of UV exposure on sirtuin expression and activity. Overall change in sirtuin levels or activity are indicated by up and down arrows. Locations of sirtuins and pathways indicate whether expression or activity has been observed in keratinocytes (epidermis) or HDF (dermis). Changes in sirtuins after prolonged UV exposure lead to fibroblast senescence and SASP, while increased MMP degrades collagen and elastin. DEJ, dermal‐epidermal junction; MMP, matrix metallopeptidase; ROS, reactive oxygen species; SASP, senescence‐associated secretory phenotype. SIRT, sirtuin; SOD, superoxide dismutase; UV, ultraviolet.

Melanocytes, located in the basal layer of the epidermis, are pigment‐producing cells that protect the skin from UVR. While few studies have investigated sirtuin involvement in melanocytes, two studies demonstrate that SIRT7 inhibits melanin production via its succinylation activity, with knockdown increasing melanin synthesis and dyspigmentation (Ma and Chang 2024; Siddiqui and Kim 2021).

## 4 Sirtuins and Aging Skin

Sirtuin expression and activity naturally decline with chronological age. In HDFs, age‐related decline of SIRT1 was seen after age 40 in healthy females (Kalfalah et al. 2014). SIRT2 declines with age in keratinocytes (Miwa et al. 2025). SIRT4, SIRT6, and SIRT7 expression was lower in aged HDFs and skin biopsies from aged volunteers compared with children and younger adults (Yi et al. 2024; Chang et al. 2020; Van Meter et al. 2014; Sharma et al. 2013; Lee, Shin, Kim, Kim, et al. 2021). SIRT7's age‐related decline is in contrast to its elevation after UVR (Lagunas‐Rangel 2022). SIRT3 decline has been demonstrated in mice and human cells, but not specifically in skin (Kwon et al. 2017; Ji et al. 2022; Lee, Shin, Kim, Kim, et al. 2021). SIRT5 is the least studied sirtuin in skin, with some evidence indicating reduced activity with age in other tissues (Kumar and Lombard 2015).

SIRT1 levels correlate with decreasing fibroblast numbers in the dermis and reduced proliferation and metabolic activity in aging fibroblasts—the imbalance in cell turnover causes ECM changes, decreasing collagen, hyaluronic acid, and elastin. In human keratinocytes, filaggrin is expressed in a SIRT1‐dependent manner, with reduced filaggrin disrupting the epidermal barrier function (Bielach‐Bazyluk et al. 2021; Blander et al. 2009).

SIRT7 knockdown in melanocytes results in increased expression of microphthalmia‐associated transcription factor, a key regulator of melanin production, suggesting a probable mechanism of increased pigmentation seen in aged skin, vitiligo, and melasma (Siddiqui and Kim 2021; Ma and Chang 2024).

In studies comparing sirtuin expression between sun‐exposed and sun‐protected skin in the same individuals, decreased SIRT1 and SIRT5 were associated with both age and UV exposure (Ding et al. 2018; Zhang et al. 2026). Of note, one study reported decreased SIRT4 in exposed skin in middle‐aged volunteers (35–52 years), and decreased SIRT6 and SIRT7 in elderly (70–83 years) versus middle‐aged skin, but no differences between exposed and unexposed skin in the elderly group for any of the sirtuins (Lee, Shin, Kim, Kim, et al. 2021). The authors suggest this may be attributed to tissue or sex differences; however, further studies are needed to resolve these discrepancies.

Aging skin is characterized by epidermal thinning, reduced elasticity, and barrier dysfunction (Furman et al. 2025). Chronologically aged human skin showing a decline in SIRT4 was associated with a thin epidermis, fewer dermal collagen fibers, and infiltration of inflammatory cells in the dermis (Yi et al. 2024).

## 5 Age‐Related Decline of the Sirtuin Substrate NAD+

Sirtuin activity depends on the availability of NAD+ as a substrate. During sirtuin‐mediated deacetylation, NAD+ is consumed, and consequently, intracellular NAD+ concentrations directly regulate sirtuins' enzymatic activity.

Multiple studies have demonstrated that NAD+ levels decrease during aging in numerous mammalian tissues, including human skin (Massudi et al. 2012; Tarragó et al. 2018; Camacho‐Pereira et al. 2016). Several mechanisms contribute to this decline, including reduced synthesis (Chini et al. 2020), increased consumption by DNA repair enzymes in response to accumulated DNA damage (Massudi et al. 2012), enhanced degradation by NADase CD38 that increases with age and chronic inflammation (Tarragó et al. 2018), and mitochondrial dysfunction and altered metabolic homeostasis (Camacho‐Pereira et al. 2016). When NAD+ levels decline, sirtuin function decreases, even if protein expression remains unchanged.

## 6 Sirtuins and the Hallmarks of Aging in Skin

Aged skin is characterized by epidermal atrophy, flattening of the rete‐ridges, degeneration of collagen and elastic fibers, and reduction of skin vasculature. Visible signs of skin aging include wrinkles, lack of firmness, decreased elasticity, and hyperpigmentation (Flament et al. 2013).

There are 13 established biological hallmarks of aging skin: genomic instability, telomere attrition, epigenetic alterations, loss of proteostasis, disabled macroautophagy, cellular senescence, mitochondrial dysfunction, deregulated nutrient sensing, stem cell exhaustion, ECM changes, chronic inflammation, altered intercellular communication, and dysbiosis (Furman et al. 2025; López‐Otín et al. 2023; Kroemer et al. 2025), with Kroemer et al. (2025) adding ECM changes and psychosocial isolation to the original 12. These hallmarks are categorized into primary, antagonistic, and integrative hallmarks, with many exacerbating or linked to other hallmarks. In this review, we focus on these 13 hallmarks and describe sirtuins' involvement in each of these processes. Literature searches (PubMed) were conducted for each sirtuin using keywords from the hallmarks of aging and limited to animal and human evidence. Nested searches identified studies in skin. No date limitations were used. The consequences of sirtuin decline on each of these hallmarks are presented, with emphasis on evidence provided in skin cells, where available (Table 2, Figure 2).

**TABLE 2:** Sirtuins and the hallmarks of aging.

Hallmark	SIRT1	SIRT2	SIRT3	SIRT4	SIRT5	SIRT6	SIRT7
Genomic instability	Non‐skin: Under oxidative stress, SIRT1‐deficient mouse ESCs show increased chromosomal fusions and translocations (Oberdoerffer et al. 2008). Skin: Downregulation of SIRT1 in human skin fibroblasts, reduced NER repair and reduced cell viability (Fan and Luo 2010).	Non‐skin: SIRT2‐deficient mice have increased DSB and chromosomal aberrations (Serrano et al. 2013).	Non‐skin: Human cells stressed with DNA‐damaging agents like UV, cause SIRT3 to be rapidly degraded, allowing transcription of several mitochondrial and stress‐related genes (Iwahara et al. 2012).			Non‐skin: Downregulation of SIRT6 reduces the crosstalk between histone modifier and chromatin remodeler, inhibiting repair and exposing DNA to further damage (Toiber et al. 2013). Skin: In HDFs, efficiency of BER correlates with both age and decline in SIRT6 expression (Xu et al. 2015).	Non‐skin: SIRT7 KO mice have impaired DSB repair and replication stress (Vazquez et al. 2016). In human cell lines, SIRT7 depletion impairs chromatin compaction during DNA‐damage response and sensitizes cells to genotoxic stresses (Li et al. 2016).
Telomere attrition	Non‐skin: SIRT1‐deficient MEFs showed significantly shorter telomeres and DNA damage at telomere ends compared with wild‐type MEFs (Palacios et al. 2010).					Non‐skin: SIRT6 depletion leads to telomere dysfunction, including end‐to‐end chromosomal fusions, premature senescence, and telomere abnormalities resembling Werner syndrome in human cells, MEFs and mice (WT and KO) (Michishita et al. 2008).	
Epigenetic alterations	Non‐skin: Loss of SIRT1 causes hyperacetylation of histones and loss of genomic stability (Wątroba et al. 2017).	Non‐skin: Loss of SIRT2 in mice induces significant defects associated with defective H4K20 methylation levels (Serrano et al. 2013).	Non‐skin: Full‐length, nuclear SIRT3 functions as an HDAC, keeping the chromatin compressed and repressing transcription (Scher et al. 2007).	Skin: In keratinocytes, loss of SIRT4 in aged cells was associated with senescence and SASP (Yi et al. 2024).	Skin: Decreased SIRT5 in human cells and in aged SIRT5 KO mice, reduced demalonylation of histones, reducing rRNA expression (Zhang et al. 2023).	Skin: Loss of SIRT6 in HDFs increases L1, genomic instability, and senescence (Van Meter et al. 2014).	Non‐skin: SIRT7 depletion in human cell lines reduced H3K122 desuccinylation, impairing chromatin compaction during DNA‐damage response, and sensitizes cells to genotoxic stresses (Li et al. 2016).
Loss of proteostasis	Non‐skin: Loss of Sirt1 leads to the accumulation of ubiquitinated proteins in cells and tissues of MEFs and SIRT1 (−/−) KO mice (Tomita et al. 2015).		Non‐skin: Loss of SIRT3 causes an imbalance of the mitochondrial membrane's potential, resulting in aggregation of mitochondrial proteins (Papa and Germain 2014).			Non‐skin: SIRT6 deletion increases protein translation, leading to reduced folding capacity and production of aggregates, as seen in human brain datasets, human and mouse cells, and KO mice (Stein et al. 2026).	
Disabled macroautophagy	Skin: SIRT1 loss increases NF‐kB activation, decreasing expression of autophagy‐associated proteins in senescent HDFs (Sung et al. 2021).	Non‐skin: Low expression of SIRT2 in NSCLC tissue was associated with lower survival rates (Wang et al. 2020). Loss of SIRT2 dysregulated autophagic/mitophagic processes in neurons and MEF cells (Liu et al. 2017).	Skin: In human keratinocytes and a psoriasis mouse model, reduced SIRT3 decreased autophagy (Yanli et al. 2023). SIRT3 inhibition reduced mitophagy biomarkers PINK1 and Parkin in DFs and skin tissue of mice (Sun et al. 2025). SIRT3 inhibition in human keratinocytes reduced AMPK‐mediated autophagy (Li et al. 2018).	Skin: In HEK and HDFs, age‐associated upregulation of SIRT4 increased the mitochondrial fusion regulator OPA1, resulting in increased mitochondrial fusion which blocked mitophagy (Lang et al. 2017).	Non‐skin: Overacylation due to SIRT5 deficiency leads to autophagy/mitophagy defects in SIRT5 KO mice (Wang, Meyer, et al. 2019).	Skin: SIRT6 knockdown in human melanoma cells modulated autophagy markers and reduced autophagosome formation (Garcia‐Peterson, Ndiaye, et al. 2017).	Non‐skin: SIRT7 deficiency inhibited autophagy in chondrocytes from osteoarthritic patients (Wu et al. 2020).
Cellular senescence	Skin: miRNAs in aged keratinocytes target SIRT1, inducing senescence (Rivetti di Val Cervo et al. 2012). Loss of SIRT1 in HDFs increases NF‐κB activity, increasing expression of inflammatory mediators (IL‐1β, IL‐6, IL‐8, and MMP‐1/‐3) (Sung et al. 2021).		Non‐skin: Loss of nuclear SIRT3 leads to irregular nuclear envelope, loss of chromatin structure, and increased senescence (Diao et al. 2021). Skin: SIRT3 inhibition in mouse DFs increased cell senescence phenotype (Sun et al. 2025). Oxidative stress lowered SIRT3/AMPK‐ controlled autophagy, resulting in senescence in NHEK (Li et al. 2018).	Skin: SIRT4 is upregulated in HDFs undergoing replicative or stress‐induced cellular senescence and in photoaged skin, due to decline in miRNA‐15b levels (Lang et al. 2016).		Non‐skin: Telomere attrition due to SIRT6 depletion leads to fibroblast senescence (Michishita et al. 2008). Skin: Aged human fibroblasts demonstrating replicative senescence have lower SIRT6 levels (Mao et al. 2012). miR‐663a and miR‐4706 that directly target the 3′‐UTR of SIRT6 are upregulated in HDFs after UVB exposure, promoting HDF senescence (Li et al. 2025).	Non‐skin: SIRT7‐deficient human cells demonstrated defective rDNA‐heterochromatin silencing and rDNA instability with excision and loss of rDNA gene copies, which induces acute senescence (Paredes et al. 2018).
Mitochondrial dysfunction	Non‐skin: Oxidative stress causes SIRT1‐dependent transcriptional deregulation in mouse ESCs (Oberdoerffer et al. 2008). Reduced SIRT1 activity increased ROS formation in the mitochondria (Poulose and Raju 2015). Skin: SIRT1 is decreased in aged HDFs via oxidative stress (Carlomosti et al. 2017). UV initially decreases SIRT1, rebound activates AMPK, reduces ROS, and inflammatory pathways (Cao et al. 2009).	Non‐skin: SIRT2‐knockout mice showed mitochondrial failure, redox imbalance, and energy depletion (Xu et al. 2023). Skin: Reduced SIRT2 in HDFs increased fission (Cha et al. 2021).	Non‐skin: Loss increases mtSOX, metabolic reprogramming, inflammation, and senescence (Wu et al. 2022; Trinh et al. 2024). Skin: UVB decreases SIRT3, decreasing ATP and SOD2 (Gan et al. 2023; Dong et al. 2012). In keratinocytes, decreased SIRT3 triggers oxidative damage, impaired mitochondrial ROS clearance, and metabolic reprogramming (Syed and Mukhtar 2013; Tao et al. 2010).	Non‐skin: Reduced SIRT4 causes increased FAO (Grabowska et al. 2017). Skin: In HEK and HDFs, age or stress‐induced upregulation of SIRT4 decreased mitochondrial respiration and increased mitochondrial ROS (Lang et al. 2017).	Non‐skin: Hypersuccinylation due to SIRT5 deficiency impairs mitochondrial respiration, FAO and GDH activity, and causes increased oxidative stress and metabolic inflexibility (Wang, Meyer, et al. 2019).	Non‐skin: SIRT6 deficiency causes an imbalance between glycolysis and mitochondrial respiration, impacting glucose homeostasis (Zhong et al. 2010).	Non‐skin: SIRT7 KO or loss causes impaired lipid metabolism and impaired mitochondrial gene transcription through its impact on GABPβ1 function (Ryu et al. 2014; Cao et al. 2021).
Deregulated nutrient sensing	Non‐skin: SIRT1 loss is associated with decreased insulin secretion, IR, T2DM, and dyslipidemia (Houtkooper et al. 2012).		Non‐skin: Mice with SIRT3 loss have fatty oxidation disorders, and metabolic syndrome (Hirschey et al. 2010). Reduced SIRT3 activity is associated with mitochondrial dysfunction, ectopic lipid accumulation, and impaired insulin signaling (Szczęsny‐Małysiak et al. 2026).	Non‐skin: SIRT4 KO mice have increased plasma insulin and FAO. Dysregulation is linked to various aging‐related disorders, including Type 2 diabetes, cardiac hypertrophy, non‐alcoholic fatty liver disease, obesity, and cancer (Szczęsny‐Małysiak et al. 2026).	Non‐skin: Altered SIRT5 activity affects insulin‐sensitive tissues by changing central carbon metabolism and redox balance, and causing metabolic inflexibility (Szczęsny‐Małysiak et al. 2026).	Non‐skin: Loss of SIRT6 leads to HIF‐1α activation, enhanced glycolysis, and increased glucose uptake that contributes to systemic metabolic imbalance, resulting in insulin resistance and diabetes (Szczęsny‐Małysiak et al. 2026). SIRT6 loss is associated with IR, T2DM, and dyslipidemia (Kane and Sinclair 2019).	Non‐skin: SIRT7‐deficient mice have hepatosteatosis, dyslipidemia (Raza et al. 2024). SIRT7 deficiency in animal models confers protection against high‐fat diet‐induced obesity, insulin resistance, and glucose intolerance, partly through enhanced energy expenditure and increased expression of insulin‐sensitizing factors such as FGF21 (Szczęsny‐Małysiak et al. 2026).
Stem cell exhaustion	Skin: SIRT1 decline increases proliferation of keratinocytes via E2F1 (Blander et al. 2009).	Skin: SIRT2 knockdown increases stem cell markers keratin 15 and keratin 19 while promoting loricrin, a terminal differentiation marker. SIRT2 knockout increased proliferation of keratinocytes (Ming et al. 2014).	Non‐skin: Loss of SIRT3 in aged HSC compromises quiescence and maintenance (Mu et al. 2021). Skin: In human epidermal keratinocytes, downregulation of SIRT3 and the subsequent rise in mtROS triggers differentiation of keratinocyte stem cells (Bause et al. 2013).	Skin: SIRT4 is transiently increased during keratinocyte differentiation (Benavente et al. 2012).	Skin: SIRT5 is transiently increased during keratinocyte differentiation (Benavente et al. 2012).	Skin: Age‐related down‐regulation of SIRT6 in aged HDFs decreases reprogramming efficiency (Sharma et al. 2013).	Non‐skin: SIRT7 deficiency results in the reduced capacity of stem cells to proliferate (Lagunas‐Rangel 2022).
ECM changes	Skin: SIRT1 knockdown in HDF increases MMP1 and MMP3, resulting in degradation of ECM collagen and elastin (Orioli and Dellambra 2018; Joo et al. 2017).					Skin: Loss of SIRT6 increases degradation of ECM collagen and elastin in HDFs (Orioli and Dellambra 2018; Joo et al. 2017). SIRT6 knockdown in HDFs inhibited COLA1 transcription and caused hyperactive NF‐κB signaling, leading to increased MMP‐1 and a decrease in dermal collagen fibrils (Baohua and Li 2012).	Skin: SIRT7 expression in aged fibroblasts is higher than in young mice at wound sites. Exosome transfer of miR‐125b from young to old mice reduced SIRT7, inhibited expression of αSMA and COL1, and increased cell migration (Xia et al. 2022).
Chronic inflammation	Non‐skin: SIRT1 knockdown in human dendritic cells increased secretion of TNFα from macrophages (Liu et al. 2015). Skin: SIRT1 inhibition increased MMP‐1 and MMP‐3 expression in HDFs (Ohguchi et al. 2010).	Non‐skin: In aged mice, SIRT2 represses activation of the NLRP3 inflammasome, induced by mitochondrial stress in mouse HSC (Luo et al. 2019). SIRT2 inhibition in mouse brain tissue increased cytokine release via upregulation of the NF‐κB pathway (Wang, Yang, et al. 2019).	Non‐skin: SIRT3 KO mice in acute lung injury model had increased neutrophils and cytokines TNF‐α, MIP‐2, and IL‐18 (Kurundkar et al. 2019). Skin: SIRT3 inhibition and KO activated inflammation via the IL‐23 axis, thereby inducing IL‐6 and TNF‐α in a psoriasis mouse model (Guo et al. 2023).			Non‐skin: SIRT6 deficiency in macrophages increased inflammation via FOXO1 stabilization (Woo et al. 2018). Skin: SIRT6 knockdown in HDFs prompts MMP release and hyperactive NF‐κB p65 signaling (Baohua and Li 2012). SIRT6 knockdown in HDFs increased NF‐_K_B (Goyarts et al. 2017).	Non‐skin: SIRT7 decline or mouse KO show an increased inflammatory response in cardiomyopathy, acute lung infections, dental disease, and age‐related neuroinflammatory conditions. Low SIRT7 promotes TGF‐β signaling, which compromises the endothelial barrier and increases inflammation (Raza et al. 2024).
Altered intercellular communication		Skin: Diminished SIRT2 activity in aged keratinocytes results in acetylation and destabilization of the tight junction protein CLDN4, disrupting barrier integrity (Miwa et al. 2025).					Non‐skin: In human kidney cells, SIRT7 and TGF‐β control each other's expression through feedback loop; deregulation causes excessive ECM deposition and fibrosis (Tang et al. 2020).
Dysbiosis	Gut microbiome: Bile acid metabolites and gut microbiota can impact SIRT1 expression (Garzon‐Escamilla et al. 2026).		Gut microbiome: SIRT3‐deficient mice exhibited increased gut inflammation, fewer beneficial bacteria, and a higher presence of pathobionts (Zhang et al. 2018).				

*Note:* Evidence of the effects of sirtuin involvement in each of the hallmarks of aging, including reports from aged cells, donors, or mouse models, or from knockout, knockdown, or inhibition studies.

Abbreviations: BER, base excision repair; ESC, embryonic stem cells; HR, homologous repair; MEF, mouse embryonic fibroblasts; MRN, Mre11‐Rad50‐Nbs1; NBS, Nijmegen Breakage Syndrome; NER, nucleotide excision repair; NHEJ, non‐homologous end joining; NLRP3, nucleotide‐binding oligomerization domain‐like receptor (NLR) family pyrin domain containing 3; NSCLC, non‐small cell lung carcinoma; SASP, senescence‐associated secretory phenotype; TERT, telomerase reverse transcriptase; TET2, ten‐eleven translocation 2; TFEB, transcription factor EB; UVR, ultraviolet radiation; XPG, Xeroderma pigmentosum group.

**FIGURE 2:**
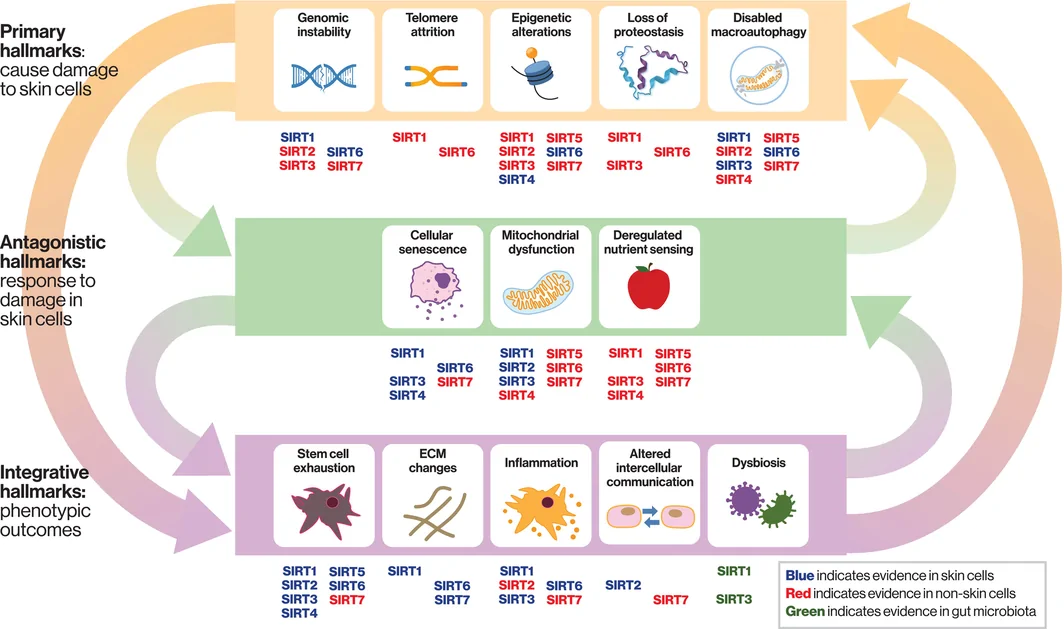
Sirtuins and the hallmarks of aging in skin. Evidence of SIRT 1–7 involvement in each hallmark due to age‐related, knockdown, or knockout changes in expression or activity. Each hallmark category feeds back to other groups, continuing the aging cycle. ECM, extracellular matrix.

### 6.1 Genomic Instability

Genomic instability is defined as the accumulation of DNA damage and impaired DNA repair mechanisms (Furman et al. 2025). These include point mutations, single‐ and double‐strand breaks (DSB), deletions, as well as chromosomal rearrangements, translocations, telomere shortening, and defects in nuclear architecture, and can occur in both nuclear and mitochondrial DNA (López‐Otín et al. 2023).

The nuclear sirtuins, SIRT1 (Oberdoerffer et al. 2008; Fan and Luo 2010), SIRT6 (Xu et al. 2015; McCord et al. 2009), and SIRT7 (Wu et al. 2018) function in DSB and point mutation repair, while SIRT3 translocates from the mitochondria to the nucleus in response to DNA damage to facilitate DNA repair (Sengupta and Haldar 2018) (Table 1, Figure 3I). These repair activities have been demonstrated in non‐skin cells, apart from SIRT1 and SIRT6. In response to UVR in human skin fibroblasts, SIRT1 deacetylates Xeroderma pigmentosum group A, an essential factor in the nucleotide excision repair (NER) pathway. Downregulation of SIRT1 results in increased sensitivity to UVR, reducing cell viability (Fan and Luo 2010). In HDFs, SIRT6 is activated by oxidative stress via JNK phosphorylation. SIRT6 then deacetylates H3K56, which recruits the chromatin remodeler SNF2h to DSBs, making the chromatin more accessible to repair factors (Van Meter et al. 2016). SIRT6 has been shown to regulate PARP1‐dependent base excision repair (BER) in HDFs, with reduced efficiency of BER correlating with both age and decline in SIRT6 expression (Xu et al. 2015).

**FIGURE 3:**
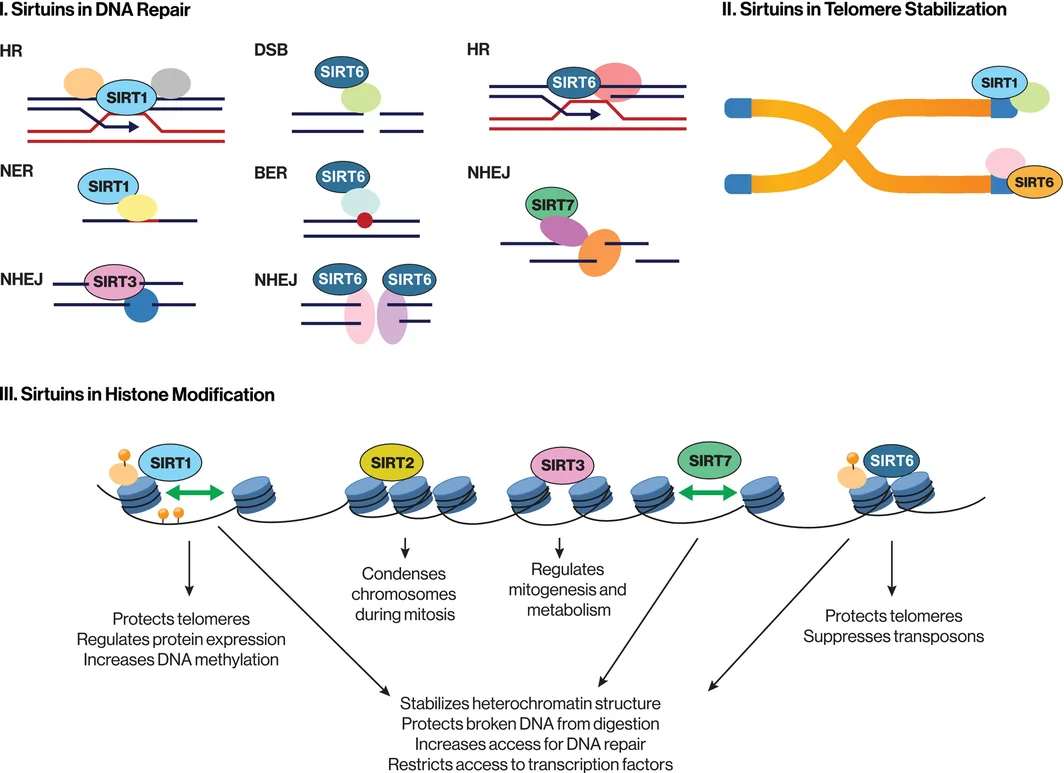
Roles of sirtuins in genomic stability and epigenetic regulation. Sirtuins involved in mechanisms of DNA repair (I), telomere stabilization (II), and histone modification (III). Sirtuins bind directly to multiple substrates to activate and provide access to repair machinery, as described in the text. BER, base excision repair; DSB, double‐strand break; HR, homologous DSB repair; NER, nucleotide excision repair; NHEJ, non‐homologous end‐joining.

Studies have shown that loss of SIRT1, SIRT2, SIRT3, SIRT6, and SIRT7 results in increased DNA damage, chromosomal abnormalities, and decreased cell viability (Table 2). Increasing SIRT1, SIRT3, and SIRT6 activity has been shown to protect dermal fibroblasts from UV‐induced DNA damage and senescence (Lee et al. 2022; Iwahara et al. 2012; Wang, Qin, et al. 2023).

DNA damage accumulates over a lifetime of exposure to exogenous stressors and spontaneous errors in replication. The efficiency of DNA repair mechanisms has also been shown to decline with age, resulting in a net increase in genetic instability (López‐Otín et al. 2023). The reported evidence indicates that the age‐related declines in sirtuins are causally linked to this increased genomic instability.

### 6.2 Telomere Attrition

Telomeres are structures comprised of repetitive nucleotide sequences at the ends of chromosomes that protect against DNA degradation, recombination, and fusion, and also prevent uncontrolled cell proliferation. Telomeres naturally shorten with age, limiting the number of times a cell can divide, eventually leading to cellular senescence and age‐associated decreased turnover, as has been observed in aged human skin biopsies (Furman et al. 2025; Pereira et al. 2019).

Both SIRT1 and SIRT6 stabilize telomeres, with SIRT6's role demonstrated in human skin (Palacios et al. 2010; Michishita et al. 2008) (Figure 3II). SIRT1's protection of telomeres and facilitation of telomere replication has been demonstrated in mouse embryonic fibroblasts (MEF) and mouse adult cells (Palacios et al. 2010). Telomerase, an enzyme complex that counteracts telomere erosion, is typically expressed in dividing stem cells (Buckingham and Klingelhutz 2011). In skin, telomerase protects telomeres in dividing keratinocyte stem cells within the basal layer of the epidermis, but is absent in somatic dermal fibroblasts and differentiated keratinocytes (Buckingham and Klingelhutz 2011). SIRT1 enhances telomerase transcription in MEFs (Palacios et al. 2010). Of note, telomerase activity increases after UVR, as well as in psoriatic epidermis (Taylor et al. 1996). SIRT6 directly impacts expression of a telomerase component and telomerase activity in hypertrophic ligamentum flavum, an age‐related fibrotic condition (Chen et al. 2021).

SIRT6's localization to telomeric chromatin in S‐phase suggests a role in regulating telomere replication. SIRT6 activates telomeres' DNA repair mechanisms in response to oxidative damage in human fibroblasts, preventing telomere loss (Michishita et al. 2008; Gao et al. 2018).

Both SIRT1 and SIRT6 depletion lead to telomere dysfunction, with SIRT1 deficiency showing shortening and increased DNA damage in telomeres (Palacios et al. 2010), while SIRT6 deficiency resulted in increased chromosomal fusions and premature senescence (Michishita et al. 2008) (Table 2). Overexpression of SIRT1 was able to attenuate age‐associated telomere shortening (Palacios et al. 2010).

Telomere length and integrity can be reduced by oxidative stress, DNA damage, or compromised repair mechanisms, ultimately leading to cellular senescence. Telomere attrition is seen in many age‐associated diseases (López‐Otín et al. 2023). In aging skin, loss of SIRT1‐supported maintenance of telomere integrity during replication and SIRT6‐supported DNA repair likely contributes to reduced renewal capacity of keratinocytes, particularly in response to UVR.

### 6.3 Epigenetic Alterations

Epigenetic changes that contribute to aging include histone modification, chromatin remodeling, DNA methylation, and altered expression of non‐coding RNAs such as miRNA (López‐Otín et al. 2023). In the skin, the extent and location of DNA methylation strongly correlate with age in HDF and keratinocytes and can be used as an epigenetic clock for chronological or biological age prediction (Horvath and Raj 2018). Both DNA methylation and histone modification can be altered by environmental stressors such as UVR (López‐Otín et al. 2023), indicating the importance of epigenetic control in maintaining cellular processes in the skin.

Sirtuins are classified as Class III histone deacetylase (HDAC) enzymes, and by definition, carry out epigenetic modifications. SIRT1, SIRT2, SIRT3, SIRT6, and SIRT7 are well documented as deacetylating one or more histones, while SIRT1 and SIRT2 also methylate histones through indirect modifications, resulting in chromatin condensation associated with mitosis and transcriptional silencing, and genomic stability, including DNA repair and telomere stabilization (Seto and Yoshida 2014; Wątroba et al. 2017) (Figure 3III). Deacetylation modification of histones by sirtuins has been observed in MEFs, mouse, and human cell lines (Yuan et al. 2009; Vaquero et al. 2004, 2006; Yang et al. 2009; Li et al. 2016).

SIRT1 can also influence histone expression, activity, and chromatin remodeling through direct DNA methylation and upregulation of DNA methyltransferases (Grabowska et al. 2017; Wątroba et al. 2017). Multiple miRNAs regulate SIRT1, with miR‐34a and its positive feedback loop with p53 the most studied, whereby p53 decreases SIRT1 and increases miR‐34a in response to DNA damage. In turn, miR‐34a decreases SIRT1, which triggers increased p53 activation, finally resulting in apoptosis. This feedback loop can be triggered by oxidative stress, DNA damage, and telomere shortening (Yamakuchi and Lowenstein 2009).

Age‐related loss of SIRT4 and SIRT6 in skin cells is associated with senescence. SIRT4 directly impacts epigenetic modulation of the ten‐eleven translocation 2 (TET2) enzyme in keratinocytes, with age‐related decline associated with senescence (Yi et al. 2024). SIRT6 directly binds to and represses the activity of long interspersed element 1 (L1) retrotransposon, a mediator of genomic instability that is epigenetically silenced in somatic cells, packaging it into transcriptionally repressive heterochromatin. However, SIRT6's association with the L1 loci is reduced in senescent HDFs, resulting in increased L1 activity. Overexpression of SIRT6 in senescent cells partially restored L1 levels to those seen in young cells (Van Meter et al. 2014).

Epigenetic alteration impacts the transcription of genes involved in mitochondrial biogenesis, inflammation, cellular senescence, DNA damage, and repair pathways. A decline in sirtuins due to age or extrinsic stressors results in an increase in acetylated histones, leading to heterochromatin structure loss and increased genomic instability, increased access to DNA damaging agents, cell cycle inhibition, chromosomal aberrations, cellular senescence, and apoptosis (Wątroba et al. 2017; López‐Otín et al. 2023).

### 6.4 Loss of Proteostasis

Proteostasis is the maintenance of correctly translated, folded, and functional proteins within the cell, and the removal of damaged proteins. Loss of proteostasis results in the accumulation of oxidized, misfolded, or glycated proteins with impaired function. Age‐related increases in oxidative stress can lead to increased protein damage, saturating the repair machinery and removal processes, such as autophagy (López‐Otín et al. 2023).

SIRT1, SIRT3, and SIRT6 are all involved in maintaining proteostasis through different processes. SIRT1 regulates protein quality control in both Hsp70‐dependent and Hsp70‐independent manners (Tomita et al. 2015). SIRT3 is a regulator of the mitochondrial unfolding response. Buildup of misfolded proteins in the mitochondria leads to proteotoxic stress and initiates the mitochondrial unfolded protein response, inducing SIRT3 expression, which initiates antioxidant defenses and autophagy (Kumar and Lombard 2015). SIRT6 regulates the translation of proteins by controlling ribosomal genes, nucleolar function, and chromatin localization (Stein et al. 2026). Loss of these sirtuins leads to increased protein aggregation due to increased protein production, reduced capacity to fold proteins correctly, and reduced autophagy (Stein et al. 2026; Kumar and Lombard 2015; Tomita et al. 2015) (Table 2).

Loss of proteostasis has been shown in aging skin fibroblasts and epidermal skin cells with both a qualitative and quantitative loss of proteasome activity (Furman et al. 2025; Gruber et al. 2020). To date, no studies have reported the effect of sirtuins on proteostasis in skin cells. The activity of SIRT1, SIRT3, and SIRT6 suggests that decreased sirtuins due to aging would cause a loss of proteostasis in skin cells, triggering aggregation and autophagy. However, the autophagy response may be compromised, resulting in increased senescence and apoptosis.

### 6.5 Disabled Autophagy

Macroautophagy or autophagy is the sequestration of cytoplasmic waste into vesicles called autophagosomes, which then fuse with lysosomes, where the contents are broken down and recycled. Waste includes protein aggregates and is therefore closely linked with proteostasis; however, cytosolic DNA, lipids, glycogens, and entire organelles are also recycled in this process (López‐Otín et al. 2023). Autophagy can be activated by caloric restriction, reduced ATP, endoplasmic reticulum stress, and hypoxia (Kim et al. 2022). These triggers, except hypoxia, involve the AMPK‐mTOR pathway, which is regulated at multiple stages by all the sirtuins, except SIRT2 (Wu et al. 2022). Evidence indicates that SIRT1, SIRT3, and SIRT6 promote mitophagy, while the remaining SIRTs inhibit or limit mitophagy (Lagunas‐Rangel 2025). However, SIRT4 promotes autophagosome‐lysosome fusion in response to nutrient deprivation or other stressors (Huang et al. 2023). SIRT2 is a negative regulator of mitophagy and autophagy, with overexpression leading to decreased expression of exosomes and autophagosome factors, which leads to increased apoptosis in stressed cells (Silva et al. 2017; Liu et al. 2017; Wang et al. 2020).

Loss of SIRT1, SIRT3, and SIRT6, and age‐related increases in SIRT4, result in dysregulated autophagy and mitophagy in skin cells (Table 2). In HDFs, SIRT1 induces autophagy through deacetylation of FOXO1 and NF‐κB (Lim et al. 2017; Sung et al. 2021). In human keratinocytes and a psoriasis mouse model, SIRT3 induced mitophagy through FOXO3/Parkin activation (Li et al. 2024; Yanli et al. 2023).

In keratinocytes, dysfunctional or insufficient autophagy can result in a senescent phenotype (Gruber et al. 2020). In senescent fibroblasts, nuclear SIRT1 becomes a substrate of the autophagy process, bound by the autophagy‐protein LC3 and subjected to cytoplasmic autophagosome‐lysosome degradation (Xu et al. 2020). SIRT1 degradation via autophagy has been observed in aging human cells and tissues and animal models (Xu et al. 2020). Similarly, SIRT7 is downregulated in an autophagy‐dependent manner, where F‐box‐only protein 7 promotes the ubiquitination and proteasomal degradation of SIRT7 upon chemically induced oxidative insult; though this has not been demonstrated in skin cells (Raza et al. 2024).

In HDFs, autophagy has been shown to decline with age. This is partly due to reduced expression of components of the autophagy machinery and partly due to the decline in inducers of autophagy, such as SIRT1 (Rubinsztein et al. 2011). Overall, the age‐related decline in sirtuins leads to disabled autophagy and mitophagy.

### 6.6 Cellular Senescence

Another hallmark of aging is the accumulation of senescent cells, i.e, cells that can no longer proliferate but are still viable and maintain metabolic activity (Wei et al. 2024). The accumulation of senescent cells is generally associated with inflammation and age‐related diseases (Xu et al. 2020). Replicative senescence occurs when cells can no longer divide due to telomere depletion, indicating that loss of SIRT1's and SIRT6's telomeric protection would increase senescence, while stress‐induced senescence occurs in response to oxidative stress and DNA damage (Wei et al. 2024). UVR causes stress‐induced cellular senescence in HDFs (Lang et al. 2016), and oxidative stress has been shown to decrease expression of all seven sirtuins in HDFs (Gilbert et al. 2023).

Aged senescent human keratinocytes and HDFs have reduced SIRT1 and SIRT6, respectively (Rivetti di Val Cervo et al. 2012; Mao et al. 2012), while decreased SIRT3 in human keratinocytes also leads to senescence (Li et al. 2018) (Table 2). Unlike other sirtuins, SIRT4 is typically upregulated in photoaged skin and in HDFs demonstrating stress‐induced and replicative senescence. This increase in SIRT4 leads to a decrease in mitochondrial functions (i.e., increase in ROS, decrease in mitochondrial potential) (Lang et al. 2016).

Senescent cells exhibit a senescence‐associated secretory phenotype (SASP), expressing and releasing cytokines, growth factors, and other auto‐ and paracrine factors; in the dermis, these are released into the ECM (Lang et al. 2016). SIRT1, SIRT6, and SIRT7 all suppress the pro‐inflammatory SASP to some degree, with this activity demonstrated in HDFs for SIRT1 via its inhibition of NF‐κB activity (Sung et al. 2021). SIRT6 also inhibits NF‐κB activity and would be expected to resist SASP development (Chang et al. 2020). Reduced autophagy increases senescence in HDFs (Sung et al. 2021). In aged cells and tissues, SIRT1 becomes an autophagy substrate, which may be one mechanism for the development of SASP (Xu et al. 2020).

With age, senescent cells accumulate in almost every skin compartment (Furman et al. 2025). Senescent keratinocytes are often found in sun‐exposed skin, while melanocytes are the primary senescent cell type in chronologically aged skin (Hughes and Bishop 2022). SASP from senescent melanocytes can limit the proliferation of surrounding basal keratinocytes (Furman et al. 2025).

Numerous other hallmarks (genomic instability, telomere attrition, loss of proteostasis, disabled autophagy, dysregulated nutrient‐sensing, and mitochondrial dysfunction) either separately or together can cause senescence; consequently, the age‐related reductions in sirtuin expression and functions all contribute to this hallmark of aging.

### 6.7 Mitochondrial Damage and Dysfunction

Sirtuins are critical for mitochondrial biogenesis, function, survival, and regulation in response to caloric restriction and damage. Nuclear SIRT1 and SIRT6 are primarily involved in regulating the expression of proteins necessary for mitochondrial biogenesis and processes (Table 1). SIRT3, SIRT4, and SIRT5 are the mitochondrial sirtuins, with all three working together to regulate multiple metabolic pathways including glycolysis, the tricarboxylic acid (TCA) cycle, fatty acid oxidation, and the urea cycle to maintain ATP levels and control the rate of respiration (Figure 4). SIRT3 and SIRT5 promote metabolic pathways, while SIRT4 acts as a negative regulator, often working in the opposing direction via its ADP‐ribosylation activity (Kumar and Lombard 2015). SIRT3 and SIRT5 reduce ROS levels, enhancing mitochondrial stability and protecting cells from apoptosis (Wu et al. 2022; Kumar and Lombard 2018; Tao et al. 2010; Wei et al. 2024). In contrast, SIRT4 inhibits glutamate metabolism, fatty acid oxidation, and amino acid catabolism, and can increase ROS levels, particularly under nutrient‐depleted conditions, which can lead to cellular senescence (Haigis et al. 2006; Ji et al. 2022; Lang et al. 2016).

**FIGURE 4:**
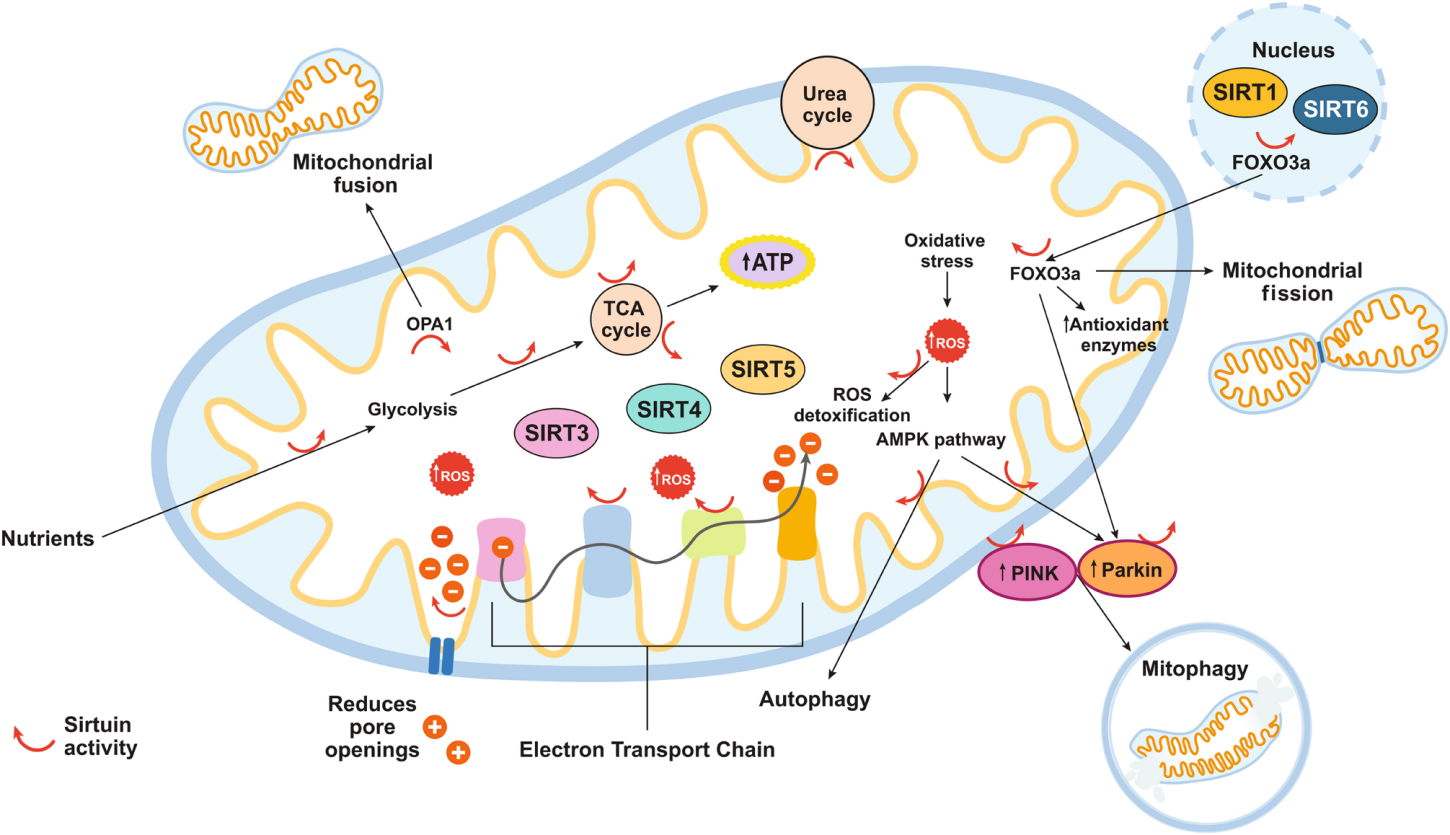
Roles of metabolic sirtuins. SIRT3, SIRT4, and SIRT5 activity regulates metabolic pathways, ATP, and electron transport chain complex, detoxifies ROS, and maintains membrane potential. When nutrients are low or oxidative stress is high, SIRTs 3–5 impact pathways for mitochondrial fusion, mitophagy, and autophagy. ROS, reactive oxygen species.

Both nuclear and mitochondrial sirtuins balance the number of mitochondria according to caloric intake and energy needs, resulting in mitochondrial fission (to increase the number of mitochondria during times of high energy needs) or mitochondrial fusion (to conserve energy) (Trinh et al. 2024; Wu et al. 2022; Verdin et al. 2010). In HDFs, cytoplasmic SIRT2 has been shown to help regulate mitochondrial division (Cha et al. 2021). The elimination of dysfunctional mitochondria via mitophagy has been discussed previously.

Mitochondrial dysfunction progresses with age, due to accumulation of mutations in nuclear and mitochondrial DNA, loss of proteostasis, increased ROS, and deregulated metabolism due to NAD+ depletion or other metabolic components (López‐Otín et al. 2023; Furman et al. 2025). In aging skin, mitochondrial dysfunction is partly caused by an imbalance between mitochondrial fusion and fission. Excessive fission causes impaired ATP production and elevated oxidative stress (Sharma et al. 2019). Notably, melanocytes create large amounts of ROS during melanogenesis, which may render these cells more susceptible to dysfunction (Furman et al. 2025). Reduced activity of all sirtuins impacts mitochondrial function, including metabolic processes and mitochondrial gene transcription. In the skin, age‐ and UV‐decreased levels of SIRT1 and SIRT3 in HDFs and keratinocytes increased oxidative stress, altered metabolic processes, and increased inflammatory pathways, while reduced SIRT2 in HDFs increased fission (Table 2).

These alterations in mitochondrial dynamics, mitochondrial biogenesis, and mitophagy are hallmarks of both intrinsic and UVR‐induced skin aging (Furman et al. 2025). In addition, the decline of NAD+ with age (Kalfalah et al. 2014) causes a pseudohypoxic state, resulting in oxidative stress that creates increased strain on sirtuin activities that combat ROS and mtSOX. Evidence indicates that the age‐related decline of sirtuins in skin would result in disrupted mitochondrial homeostasis, increased ROS, loss of ATP, and an accumulation of fragmented cells due to inefficient mitophagy. Together, these effects would put cells on the path to senescence or apoptosis.

### 6.8 Deregulated Nutrient‐Sensing

This hallmark is closely linked to metabolic dysfunction. A network of different pathways participates in the detection and processing of nutrients in the cell, each of which adapts and reacts constantly in response to nutrient supply, caloric restriction, or stress (López‐Otín et al. 2023). Adequate macronutrients and micronutrients are required for the health of all cells, including skin cells (Furman et al. 2025). In general, caloric restriction is associated with cell longevity, while anabolic signaling accelerates aging (López‐Otín et al. 2023; Lagunas‐Rangel 2022).

Sirtuins are central to the crosstalk between nutrient‐sensing pathways (insulin/IGF‐1, mTOR, AMPK) (Haigis et al. 2006; Trinh et al. 2024; Lagunas‐Rangel 2022), with SIRT1, SIRT3, and SIRT6 activated in nutrient scarcity (López‐Otín et al. 2023; Grabowska et al. 2017). SIRT1 promotes metabolic flexibility, allowing cells to shift from glucose utilization toward increased fatty acid oxidation and mitochondrial respiration during energy deprivation. SIRT3 is strongly induced during fasting and caloric restriction, where it enhances fatty acid oxidation, the TCA cycle, and antioxidant defense systems such as SOD2 (Szczęsny‐Małysiak et al. 2026). SIRT4 generally counterbalances catabolic metabolism by suppressing fatty acid oxidation while promoting lipid anabolism and modulating amino acid‐driven insulin secretion, thereby contributing to energy conservation (Haigis et al. 2006). SIRT5 suppresses glucose metabolism in response to changes in metabolic substrate availability (Park et al. 2013). SIRT6 represses glycolytic gene expression under conditions of energy stress. SIRT7 senses glucose with low levels, resulting in reduced SIRT7‐mediated mitochondrial biogenesis, cellular respiration, and gluconeogenesis, and cAMP signaling (Lagunas‐Rangel 2022; Szczęsny‐Małysiak et al. 2026). Loss of sirtuin functionality results in metabolic dysfunction, with several symptoms of metabolic syndrome associated with multiple sirtuins, including insulin resistance, dyslipidemia, and obesity (Table 2).

In skin, efficient nutrient‐sensing results in improved cell survival and less oxidative damage from nutrient excess (Furman et al. 2025). Energy restrictions increase the NAD+/NADH ratio, which activates SIRT1, leading to differentiation of keratinocytes and extended life of dermal fibroblasts (Gerhart‐Hines et al. 2007; Orioli and Dellambra 2018). Aside from the link between deregulated nutrient‐sensing and dysfunctional mitochondria, age‐related changes to nutrient‐sensing capability can cause excessive anabolic signaling, reduced autophagy, and uncontrolled inflammation (López‐Otín et al. 2023). In aged skin, fibroblasts produce less IGF‐1, reducing IGFR signaling in keratinocytes, thereby attenuating the cell's ability to combat UVB‐induced DNA damage. Elevation of the mTOR/Akt signaling pathway increases NF‐κB activation in both intrinsic and photo‐induced skin aging (Choi et al. 2016). SIRT1, SIRT3, and SIRT6 all impact the mTOR‐AMPK pathway by regulating blood glucose levels and oxidative stress (Chang et al. 2020; Wu et al. 2022). Though specific studies of skin cells and nutrient‐sensing are lacking, age‐related changes in sirtuins would be expected to alter energy‐sensing and metabolic regulation in skin.

### 6.9 Stem Cell Exhaustion

Skin barrier integrity relies on the continuous supply of new cells generated from somatic stem cells in the basal layer of the epidermis (López‐Otín et al. 2023). These stem cells include those in the interfollicular epidermis, hair follicles, and melanocytes (Furman et al. 2025). Moreover, tissue's ability to repair itself can sometimes require de‐differentiation of cells to a more stem cell‐like state (López‐Otín et al. 2023).

Stem cell exhaustion results from the loss of cellular plasticity required for tissue repair. Adult stem cell exhaustion, via a reduction in number or activity, results in cellular senescence and signs of premature aging (hair graying), changes in skin elasticity (loss of dermal plumpness), thinning epidermis, and compromised overall architecture (weakening of the dermal‐epidermal junction [DEJ]). In addition, stem cell loss results in reduced production of collagen in the ECM (Furman et al. 2025).

Stem cell maintenance is affected by genomic integrity, telomere length, Wnt signaling, regulated autophagy, and inflammation, all of which can be impacted by age‐related decline in sirtuins (Rubinsztein et al. 2011; Mu et al. 2020, 2021; Luo et al. 2019). Sirtuins sustain stem cell populations by protecting them from damage and keeping them metabolically youthful.

In vitro studies have demonstrated that multiple sirtuins are involved in the regulation of keratinocyte stem cell proliferation and differentiation (Wang, Yang, et al. 2019; Ming et al. 2014; Blander et al. 2009; Bause et al. 2013) (Table 2). SIRT1 regulates keratinocyte stem cell self‐renewal and differentiation, with overexpression inhibiting keratinocyte proliferation, thereby increasing differentiation, while underexpression increased proliferation (Blander et al. 2009). Reduced SIRT2 increases keratinocyte proliferation (Ming et al. 2014). SIRT3's anti‐mtROS activity suppresses stem cell differentiation, with loss compromising quiescence and triggering differentiation in human epidermal keratinocytes (Bause et al. 2013). Both SIRT4 and SIRT5 show a transient increase in expression in a simulated differentiation of human keratinocytes experiment, although their exact role in stem cell maintenance has not been determined (Benavente et al. 2012). In a psoriasis model using human epidermal keratinocytes, SIRT5 decreased cell proliferation and inflammation and improved barrier function via suppression of ERK/STAT3 signaling (Wang, He, and Shi 2023). While not shown in keratinocytes, SIRT6 improves reprogramming efficiency of HDFs (Sharma et al. 2013). SIRT7 maintains quiescence of stem cells in multiple tissues, while deficiency results in the reduced capacity of stem cells to proliferate (Lagunas‐Rangel 2022).

Sirtuin deficiency due to aging would result in dysregulation of keratinocyte stem cell maintenance (Bause et al. 2013; Ming et al. 2014), reducing the skin's regenerative and repair capacity and increasing senescence and inflammation.

### 6.10 Extracellular Matrix Changes

The ECM is a complex, non‐cellular network of proteins including collagen, fibronectin and elastin, and glycans, such as hyaluronic acid (Orioli and Dellambra 2018). The ECM is linked to mitochondrial homeostasis, cellular senescence, stem cell exhaustion, proteostasis, inflammation, and intracellular communication (Kroemer et al. 2025; Furman et al. 2025). In skin, the ECM is responsible for elasticity and firmness of the skin's texture and appearance, which progressively decline with age, or in response to UV‐mediated photoaging (Kroemer et al. 2025; Garcia‐Peterson, Wilking‐Busch, et al. 2017). With age, the ECM experiences an increasing number of SASP‐secreting fibroblasts and melanocytes, a decrease in collagens (Type I, III and VII), a decline in elastic fiber remodeling, and increases in pro‐inflammatory mediators (Furman et al. 2025) (Figure 5). The DEJ layer (or rete ridges), with its naturally corrugated structure, flattens with age and becomes thinner (Aleemardani et al. 2021). In the skin, SIRT1 and SIRT6 inhibit collagen degradation by suppressing inflammation (Orioli and Dellambra 2018). In HDFs, reduced SIRT1 and SIRT6 elevate MMP‐1, which hydrolyzes collagen and accelerates the degradation of ECM components (Ohguchi et al. 2010; Orioli and Dellambra 2018) (Table 2).

**FIGURE 5:**
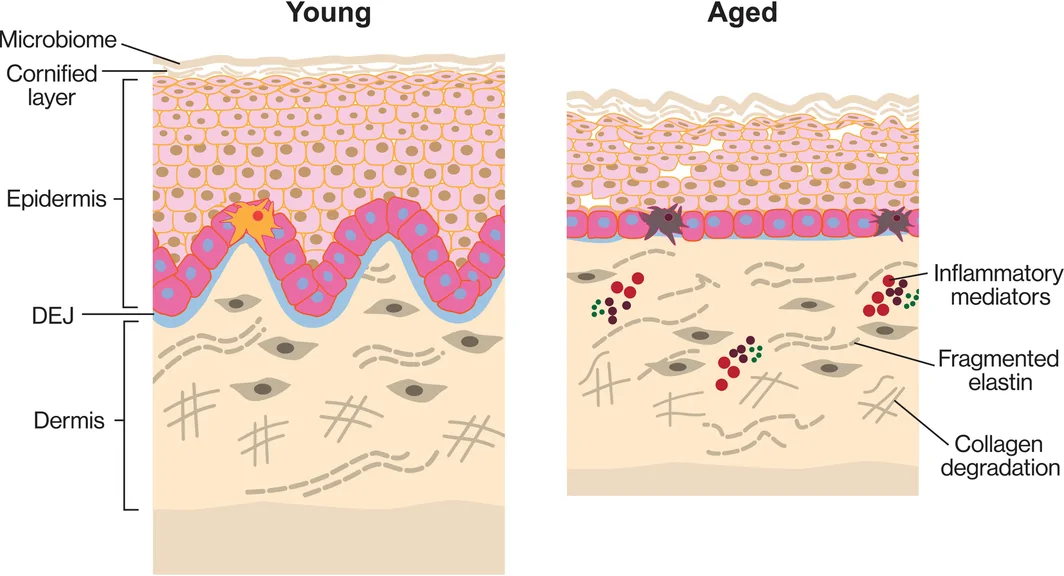
The ECM in young and aged skin. Youthful skin has a dense ECM, while aged skin is thinner, less dense, and contains SASP‐secreting fibroblasts and melanocytes, pro‐inflammatory mediators, and less collagen and elastin. DEJ, dermal‐epidermal junction; ECM, extracellular matrix.

The impact of sirtuin decline due to age or photoaging would contribute to loss of collagen and elastin in the ECM, manifesting as wrinkles and loss of plumpness, making ECM changes the most visible of the hallmarks. Moreover, the loss of sirtuins results in the ECM containing an increased number of senescent cells, SASP, and other inflammatory mediators.

### 6.11 Inflammation

Chronic age‐related inflammation, or “inflammaging,” accelerates many of the hallmarks of aging in skin, with the release of cytokines and chemokines reducing the regenerative ability of epidermal stem cells and rendering HDFs unable to maintain tissue homeostasis. Keratinocytes act as environmental stress sensors, and skin cells in general release pro‐inflammatory mediators when exposed to extrinsic insults (Furman et al. 2025).

Sirtuins play an integral role in regulating molecules central to inflammatory pathways (Wu et al. 2022). A key regulator of inflammation is NF‐κB. SIRT1 deacetylates the RelA/p65 subunit of NF‐κB in human cell lines (Yeung et al. 2004), while SIRT6 deacetylates the histone H3K9 in the promoter region of NF‐κB target genes, suppressing inflammation (Wu et al. 2022). Age‐associated NAD+ and SIRT3 decline impacts transcription of NF‐κB and FOXO family members, exacerbating inflammation susceptibility (Trinh et al. 2024; Zhou et al. 2022). Inflammatory diseases can activate the NLRP3 inflammasome, a downstream consequence of NF‐κB activation. SIRT1 and SIRT3 have been shown to inhibit NLRP3 activity via different mechanisms. SIRT7 appears to both inhibit and promote inflammation via the NF‐κB pathway, but further studies are required to uncover these mechanisms (Wu et al. 2022).

In vitro knockdown of sirtuins elevates inflammatory activity (Table 2). Suppression of SIRT1 and SIRT6 increases MMP levels in HDFs, which can degrade the ECM (Ohguchi et al. 2010; Baohua and Li 2012), while SIRT3 is downregulated in macrophages isolated from psoriatic lesions, resulting in IL‐23 axis inflammation (Guo et al. 2023).

Studies on sirtuins and inflammation in the skin are limited. In most cases, evidence of sirtuins' roles in regulating inflammatory mediators, cytokines, and the NF‐κB pathway suggests anti‐inflammatory actions (Wu et al. 2022). Reduced sirtuin activity would be expected to exacerbate inflammatory pathways; however, additional research is needed in this area.

### 6.12 Altered Intercellular Communication

This hallmark of aging refers to the decline in management of external stressors due to age‐related changes in paracrine and endocrine signaling. In aging skin, we see diminished cell‐to‐cell communication via gap junctions, inflammaging (particularly with the increase in senescent cells expressing SASP), and ECM remodeling, all contributing to altered intercellular communication (Jin et al. 2023).

In the skin, SIRT2 inhibits cargo loading and release of extracellular vesicles, which may prevent pro‐inflammatory or senescent signals (Lee et al. 2019). Moreover, diminished SIRT2 activity disrupts intercellular binding and barrier integrity (Miwa et al. 2025). While research on sirtuins in this area is scarce, SIRT7 has been shown to increase the release of signals for cell migration (Tang et al. 2020; Xia et al. 2022). Increases in the primary and antagonistic hallmarks of aging, with age‐related decline in sirtuins, would be expected to alter intercellular communication in the skin, particularly in relation to its ability to signal repair of wounds and other external insults, maintain the ECM, and sustain barrier integrity.

### 6.13 Dysbiosis

Typically, dysbiosis refers to changes in the gut microbiome, which occur with aging due to changes in metabolic pathways and disease (López‐Otín et al. 2023). In mouse studies, several sirtuins appear to impact the gut microbiome through maintaining the intestinal barrier, while microbiota and their metabolites can impact sirtuin activity (Garzon‐Escamilla et al. 2026). While this phenomenon has not been researched relative to sirtuins in the skin, the skin microbiome is critical to aging, with many age‐dependent intrinsic changes and extrinsic exposures (pH, sebum, immune functions, nutrients, sweat, UVR, temperature, humidity, cosmetics, air pollution) destabilizing the shared ecosystem between the skin and its flora. Altered microbiota can influence immunity, antimicrobial defense, and regulation of the inflammatory response in skin (Furman et al. 2025). Therefore, age‐related dysbiosis would be expected to result in increased inflammation and a weakened skin barrier, with a bidirectional relationship between sirtuin loss and skin microbiota.

## 7 Summary

Sirtuins are integral to the homeostasis of skin, providing protection and healing from UVR and other environmental insults, maintaining the pool of epidermal stem cells, and regulating keratinocyte differentiation, all while contributing to genomic integrity and metabolic function within cells, and the overall structure and composition of the skin barrier. Sirtuins decline with chronological age, with UV exposure also impacting their activities, resulting in a loss of this homeostasis. The decline in sirtuin activity impacts the majority of hallmarks of aging in skin, either directly or due to the integrated nature of the hallmarks, contributing to visible signs of aging including wrinkles, skin thinning, and loss of elasticity.

## Author Contributions

All authors conducted literature searches and analysis on each sirtuin using the hallmarks of aging as a framework. These were collaboratively developed into an initial draft. All authors reviewed and edited subsequent drafts.

## Funding

Preparation of this manuscript was funded by The Estée Lauder Companies, Melville, NY.

## Conflicts of Interest

All authors are employees of The Estée Lauder Companies.

## Data Availability

Data sharing not applicable to this article as no datasets were generated or analysed during the current study.
